# Comparison of Telephone and Video Telehealth Consultations: Systematic Review

**DOI:** 10.2196/49942

**Published:** 2023-11-17

**Authors:** Oyungerel Byambasuren, Hannah Greenwood, Mina Bakhit, Tiffany Atkins, Justin Clark, Anna Mae Scott, Paul Glasziou

**Affiliations:** 1 Institute for Evidence-Based Healthcare Bond University Robina Australia

**Keywords:** telehealth, telemedicine, telerehabilitation, systematic review, effectiveness

## Abstract

**Background:**

Telehealth has been used for health care delivery for decades, but the COVID-19 pandemic greatly accelerated the uptake of telehealth in many care settings globally. However, few studies have carried out a direct comparison among different telehealth modalities, with very few studies having compared the effectiveness of telephone and video telehealth modalities.

**Objective:**

This study aimed to identify and synthesize randomized controlled trials (RCTs) comparing synchronous telehealth consultations delivered by telephone and those conducted by video with outcomes such as clinical effectiveness, patient safety, cost-effectiveness, and patient and clinician satisfaction with care.

**Methods:**

PubMed (MEDLINE), Embase, and CENTRAL were searched via the Cochrane Library from inception until February 10, 2023, for RCTs without any language restriction. Forward and backward citation searches were conducted on included RCTs. The Cochrane Risk of Bias 2 tool was used to assess the quality of the studies. We included studies carried out in any health setting—involving all types of outpatient cohorts and all types of health care providers—that compared synchronous video consultations directly with telephone consultations and reported outcomes specified in the objective. We excluded studies of clinician-to-clinician telehealth consults, hospitalized patients, and asynchronous consultations.

**Results:**

Sixteen RCTs—10 in the United States, 3 in the United Kingdom, 2 in Canada, and 1 in Australia involving 1719 participants—were included in the qualitative and quantitative analyses. Most of the telehealth interventions were for hospital-based outpatient follow-ups, monitoring, and rehabilitation (n=13). The 3 studies that were conducted in the community all focused on smoking cessation. In half of the studies, nurses delivered the care (n=8). Almost all included studies had high or unclear risk of bias, mainly due to bias in the randomization process and selection of reported results. The trials found no substantial differences between telephone and video telehealth consultations with regard to clinical effectiveness, patient satisfaction, and health care use (cost-effectiveness) outcomes. None of the studies reported on patient safety or adverse events. We did not find any study on telehealth interventions for diagnosis, initiating new treatment, or those conducted in a primary care setting.

**Conclusions:**

Based on a small set of diverse trials, we found no notable differences between telephone and video consultations for the management of patients with an established diagnosis. There is also a significant lack of telehealth research in primary care settings despite its high uptake.

## Introduction

Telehealth (the provision of health care via telephone or video) has been used for health care delivery for decades, but the COVID-19 pandemic greatly accelerated the uptake of telehealth in many care settings globally [1]. In Australia, following a rapid national policy response to the COVID-19 pandemic, uptake of telehealth consultations increased sharply and effectively [1]. In state of Victoria, telehealth consultations with general practitioners increased from 0% before the pandemic to 55% by August 2020 and tapered off to stay around 30% thereafter [2]. The most common types of consultations through telehealth were management of chronic conditions, mental health, and medication, posttest and postdischarge follow-ups, and new patient consultations [3].

Previous systematic reviews of telehealth versus face-to-face consultations found no evidence of difference in outcomes of clinical effectiveness, patient satisfaction, and cost in many areas including mental health and primary care [4,5]. However, few studies have made direct comparisons between different telehealth modalities, with very few studies having compared the effectiveness of telephone versus video telehealth modalities. Studies that have examined this are generally narrowly focused on specific care providers such as nurses [6], or specific conditions such as chronic conditions [7], with no available systematic reviews that have compared telephone and video telehealth across a variety of care settings.

Given the now widespread use of telehealth and the predominance of telephone over video consultations [1], it is important to compare the effectiveness and acceptability of telehealth delivered via telephone to video. We therefore aimed to identify, assess the quality of, and synthesize randomized controlled trials (RCTs) that compare synchronous telephone and video provision of care.

## Methods

### Study Design

The systematic review was reported in compliance with the PRISMA (Preferred Reporting Items for Systematic Reviews and Meta-Analyses) statement [8]. The protocol was developed prospectively and is available through the Open Science Framework [9]. We used the methodology of completing a full systematic review in 2 weeks herein [10]. This systematic review was conducted as part of a series of evidence syntheses evaluating evidence for the effectiveness of telehealth for the Australian Government Department of Health and Aged Care.

### Inclusion and Exclusion Criteria

We included RCTs of any design, including parallel, cluster, crossover, factorial, or mixed, that included more than 10 participants and directly compared telephone consultations with video telehealth consultations. All other study designs (nonrandomized trials, observational studies, and qualitative-only studies), reviews (eg, literature, scoping, etc), commentaries, or opinion pieces were excluded.

### Participants

We included studies with participants of any age, gender, care setting, or health condition. Studies conducted in a tertiary care setting (with in-hospital patients) were excluded. However, studies involving patients discharged from hospital and undergoing care by a primary or allied health providers were included. Care providers could include, but were not limited to, general practitioners, allied health care providers, nurse practitioners, midwives, and specialist physicians (eg, psychiatrists, dermatologists, and rheumatologists). Telehealth consultations between patients and clinicians were included; clinician-to-clinician consultations not involving patients were excluded.

### Interventions

We included studies that evaluated the effectiveness of real-time (synchronous or “live”) consultations via telephone calls, including diagnosis, treatment and follow-up. Consultations involving asynchronous provision of care (eg, store and forward of patient-generated data) were excluded. Studies evaluating the following interventions were also excluded: mobile apps, virtual reality, SMS text messages (eg, reminders), web-based platforms (eg, information and support systems and chat-based services), and studies of novel (nonstandard) interventions. Consultations could include single or multiple episodes of care, but the compared groups had to receive similar care in terms of frequency, duration, and health care provider.

### Comparators

We included comparators that evaluated the effectiveness of real-time (synchronous) consultations via video on any device type (eg, mobile or desktop computer), including diagnosis, treatment, and follow-up. We included only direct comparisons between telephone and video telehealth consultations; indirect comparisons (of video to face-to-face care or telephone to face-to-face care) were excluded.

### Outcomes

We included studies that reported on our primary outcome of interest, which was clinical effectiveness (details depended on condition/clinical area), and secondary outcomes, which were patient safety, cost-effectiveness, and patient and clinician satisfaction with care. For diagnostic accuracy studies, the outcomes would include comparative accuracy of diagnosis for telephone versus video telehealth care.

### Search Strategy

PubMed (MEDLINE), Embase, and CENTRAL were searched via the Cochrane Library (which includes ClinicalTrials.gov and the World Health Organization’s International Clinical Trial Registry Platform) from inception until February 10, 2023. All search strategies are provided in Multimedia Appendix 1. Forward and backward citation analysis was conducted on included RCTs to ensure that all relevant studies have been identified.

### Study Restrictions

We did not impose restriction by language (ie, if the article met the inclusion criteria but was published in a language other than English, it was includable) or date. We only included studies that were published in full. We excluded publications available as abstracts only (eg, conference abstracts) with no additional results or information available about the study’s results (eg, from a clinical trial registry record).

### Study Selection and Screening

Following deduplication of the search results, review authors (OB and HG) independently screened the titles and abstracts, and full-text articles for inclusion. Any disagreements were resolved through discussion or by consulting a third author (PG). Two authors (MB and TA) screened trial database records. A list of studies excluded at the full-text stage are provided in Multimedia Appendix 2.

### Data Extraction

Review authors (OB, HG, and MB) independently extracted the data on study characteristics and methods, participants, interventions and comparators, and primary and secondary outcomes. Discrepancies in data extraction were resolved by consensus or by referring to another author.

### Assessment of Risk of Bias

The risk of bias of included RCTs was assessed independently by 2 authors (MB and TA) using the Cochrane Risk of Bias 2 Tool [11]. Five domains were assessed: bias arising from the randomization process, bias due to deviations from intended intervention, bias due to missing outcome data, bias in the measurement of the outcome, and bias in the selection of the reported results. Bias was graded as low, high, or as having some concerns. In our protocol, we stated that we would use the original Cochrane Risk of Bias tool, but during the conduct of the review, we decided to use the updated Risk of Bias 2 tool due to its recency.

### Data Analysis

RevMan 5 (version 5.4; Cochrane) was used to calculate the intervention’s effect. When appropriate, we meta-analyzed studies using either odds ratios or standardized mean differences using a random-effects model. We used odds ratios for results reporting the number of patients with an event (eg, stopped smoking) and standardized mean difference for continuous outcomes (eg, depression). Study outcomes were compared and subgrouped by follow-up duration where relevant. We relied on the RCT design to ensure that the intervention and control groups were homogenous at baseline. Due to very short time lines, we did not attempt to contact investigators or study sponsors to provide missing data.

## Results

**Figure 1:**
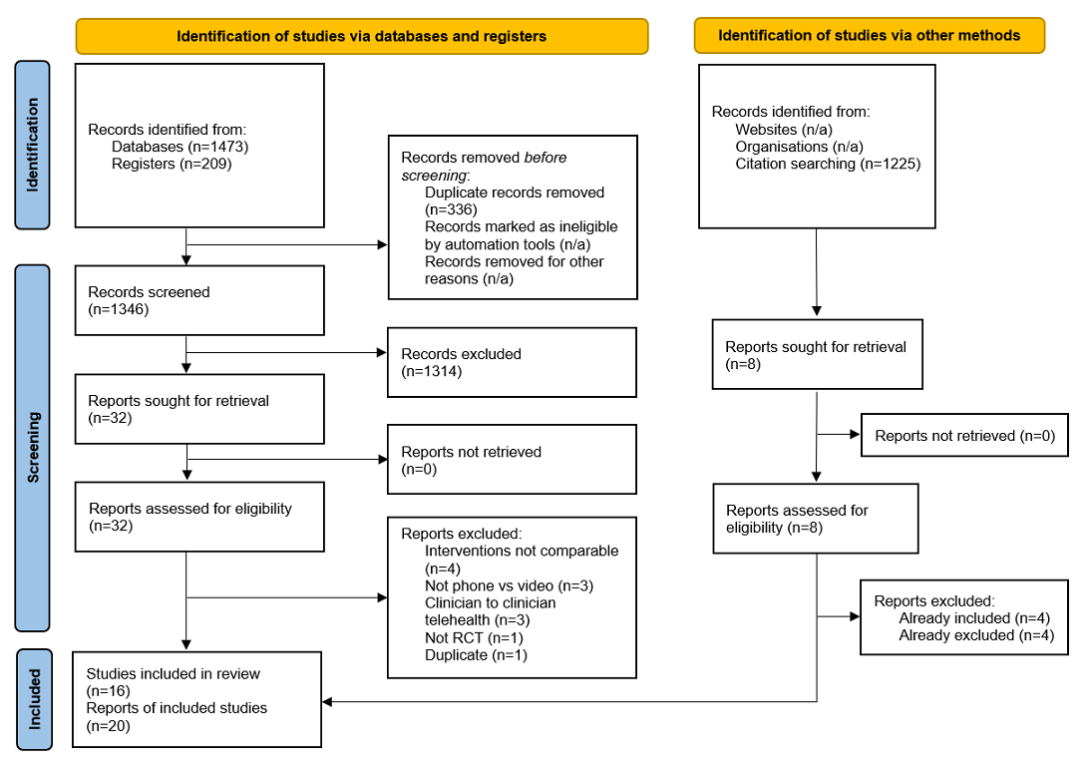
PRISMA (Preferred Reporting Items for Systematic Reviews and Meta-Analyses) flowchart. n/a: not applicable;.

We screened the titles and abstracts of 2571 articles, which included 1473 references from databases, 1225 references from citation searching, and 209 from clinical trial registries. Of the total of 40 full-text articles screened, 16 RCTs in 20 publications comprising 1719 participants were included in the final review (Figure 1). A list of excluded studies is provided in Multimedia Appendix 2.

Characteristics of the included studies are shown in Table 1. Ten studies were conducted in the United States [12-24], 3 in the United Kingdom [25-28], 2 in Canada [29,30], and 1 in Australia [31]. The majority of the telehealth interventions involved hospital-based outpatient follow-ups, monitoring, and rehabilitation (n=13) [12-17,20-30]. The other 3 studies that were conducted in the community setting were all smoking cessation studies [18,19,31]. We found no studies on telehealth comparisons for diagnosis, initiation of new treatment, or in primary care. Nine studies had a 3-arm design that compared video and telephone interventions with either treatment as usual, waitlist control, or minimal information (ie, pamphlet) [12-14,16,17,20-23,26,27,31]. Four studies involved patients’ carers [15,26-28,30]. Interventions were delivered by nurses in 8 studies [13-17,22-25,30], counselors, or therapists in 4 studies [12,18,19,31], specialist clinicians in 3 studies [20,27,28], and a physiotherapist in 1 study [29]. None of the included studies reported on clinician satisfaction, patient safety, or adverse events.

**Table 1:** Characteristics of included randomized controlled trials (RCTs).

Study; year (country)	RCT design	Follow-up duration	Total participants (phone and video), n (n, n)	Population	Intervention and comparator	Reported relevant outcomes
Byaruhanga et al [31]; 2021 (Australia)	Parallel 3-arm	4 months	699 (229, 201)	Smokers older than 18 years, who live in rural and remote areas, with access to a telephone, internet, and email	Up to 6 sessions with 15-minute smoking cessation video counseling delivered by smoking cessation advisors via video communication technology (eg, Skype) vs that delivered via telephone	7-day point prevalence abstinence, prolonged abstinence, and quit attempts
Cacioppo et al [12]; 2021 (United States)	Parallel 3-arm	6 months	119 (37, 38)	Patients with cancer who speak English and are eligible for cancer genetic testing	One session of genetic counseling by genetic counselors via HIPAA^a^-compliant videoconferencing software or telephone at the oncology clinic in addition to generic information flyers	Genetic counseling service uptake and satisfaction with telemedicine
Chambers et al [25]; 2006 (United Kingdom)	Parallel 2-arm	12 months	30 (15, 15)	Patients receiving parenteral nutrition	Standard care and follow-up according to usual protocols, with videophone or telephone with the nutrition nurse specialist: weekly for 1 month, fortnightly for 1 month, once a monthly for 4 months, and quarterly for the rest of the study	In-patient days
Egner et al [13]; 2003 (United States)	Parallel 3-arm	24 months	27 (11, 9)	Patients with multiple sclerosis who had a recent functional setback in disease process and with an Expanded Disability Status Scale score of ≥7	Structured at-home education and counseling session delivered via video or telephone by a rehabilitation nurse	Depression, fatigue, and health-related quality of life
Fincher et al [14]; 2009 (United States)	Parallel 3-arm	One-off intervention and outcome survey	75 (25, 25)	Patients with Parkinson disease who take ≥3 medications and have access to and are able to regularly use telephones and purpose-built videophones	20-minute standardized Parkinson disease medication and counseling sessions by a nurse via videophone or telephone	Patient satisfaction
Hastings et al [15]; 2021 (United States)	Parallel 2-arm	3 and a half months	40 dyads (20, 20)	Veterans aged 65 years or older with complex medical conditions and suspected mild cognitive impairment, and their care partners	12-week care management intervention: monthly video or telephone calls from a study nurse covering medication management, cardiovascular disease risk reduction, physical activity, and sleep behaviors	Feasibility, acceptability, and usability
Jerant et al^b^ [16]; 2001 (United States)	Parallel 3-arm	12 months	37 (12, 13)	Patients with congestive heart failure aged 40 years or older who speak English	Home telecare delivered via a 2-way videoconference device with an integrated electronic stethoscope or nurse telephone calls	Health care costs and patient satisfaction
Kim et al [18]; 2018 (United States)	Parallel 2-arm	6 months	42 (21, 21)	18-75–year-old women living with HIV who smoke ≥5 cigarettes per day, have smartphones, speak English, and are willing to set a quit date within 4 weeks from the first session	8 weekly counseling sessions (10-30 minutes) by a counselor for smoking cessation conducted via video or telephone calls along with open-label nicotine patches (also for 8 weeks)	Biochemically verified 2- and 6-month abstinence
Kim et al [19]; 2016 (United States)	Parallel 2-arm	3 months	49 (25, 24)	18-65–year-old Korean American women who had smoked ≥10 cigarettes per day for the last 6 months, who have access to video calls, without contraindication to nicotine patches, not pregnant or lactating, and are willing to set a quit date within 4 weeks from the baseline assessment	8 weekly counseling sessions (30 minutes) conducted by therapists for a deep culturally adapted smoking cessation intervention by video or a telephone call app along with open-label nicotine patches (also for 8 weeks). Self-help materials and family coaching was provided twice before and after the quit day	Biochemically verified and self-reported 3-month abstinence
Kingery et al^c^ [20]; 2021 (United States) and Manjunath et al [21]; 2021 (United States)	Parallel 3-arm	One-off intervention and outcome survey	2551 (119, 71)	Outpatient orthopedic surgery patients	Video or telephone follow-up call by the surgeon	Patient satisfaction
McCrossan et al^d^ [27]; 2012 (United Kingdom) and Ashley et al [26]; 2015 (United Kingdom)	Parallel 3-arm	41 months	83 (24, 35)	Infants with major congenital heart disease and their carers	Videoconferencing or telephone support with a clinician weekly or twice-weekly and urgently if needed	Health care resource use and inpatient days
Morgan et al [28]; 2008 (United Kingdom)	Parallel 2-arm	1.5 months	30 (14, 16)	Infants with major congenital heart disease and their carers	Home monitoring via videoconferencing or telephone calls following discharge from hospital, started twice-weekly and then as needed by physicians	Anxiety levels of families
Phillips et al [22]; 2001 (United States)	Parallel 3-arm	12 months	111 (36, 36)	18-60–year-old patients with newly acquired spinal cord injury	Individual educational rehabilitation sessions with a nurse via video or telephone calls once a week for 5 weeks, and then fortnightly for 1 month	Depression, quality of life, and annual hospital days
Renard et al [29]; 2022 (Canada)	Parallel 2-arm	—^e^	20 (10, 10)	Rehabilitation patients with nonurgent conditions who have access to the internet or a computer, who can follow instructions for exercises at home	Up to 6 sessions of videoconference or telephone follow-ups with a physiotherapist	Qualitative analysis of feasibility, clinical effectiveness, and patient satisfaction
Wakefield et al^f^ [24]; 2008 (United States) and Wakefield et al [23]; 2009 (United States)	Parallel 3-arm	12 months	148 (47, 52)	Patients with heart failure	Home monitoring via a purpose-built videophone or telephone 3 times in the first week after discharge, and then weekly for 11 weeks (14 contacts over 3 months by the study nurse)	6-month mortality, self-efficacy, and satisfaction with care
Young et al [30]; 2007 (Canada)	Parallel 2-arm	1.5 months	43 dyads (22, 21)	Pediatric orthopedic surgery patients and their caregivers	Follow-up using a purpose-built videophone or telephone post discharge on day 3 and as needed for 6 weeks by an orthopedic clinic nurse	Qualitative exploration of families’ experiences

^a^HIPAA: Health Insurance Portability and Accountability Act.

^b^Same studies that published their results in multiple publications.

^c^Same studies that published their results in multiple publications.

^d^Same studies that published their results in multiple publications.

^e^Not available.

^f^Same studies that published their results in multiple publications.

### Risk of Bias

**Figure 2:**
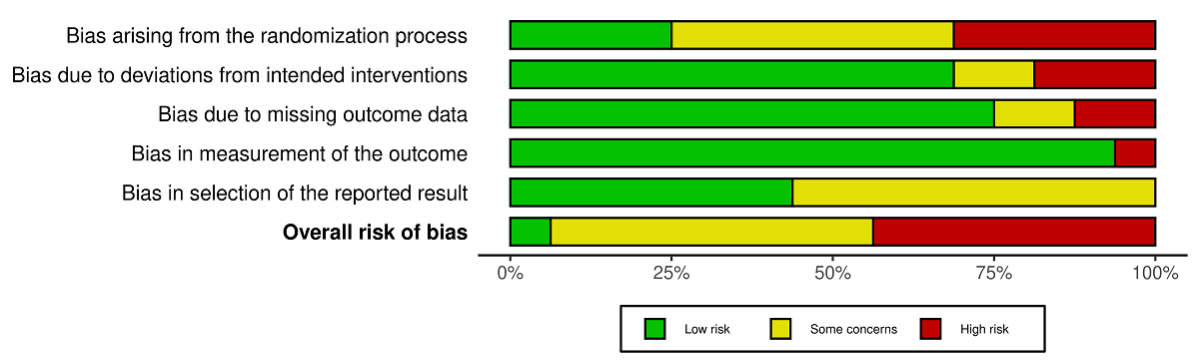
Risk of bias of included studies.

Overall, most studies had a high risk of bias or some concerns, mostly in 2 domains: randomization processes were not clearly reported in 12 studies, and we could not clearly determine bias in the selection of reported results in 9 studies. Bias due to deviations from intended interventions, missing outcome data, and bias in measurement of the outcomes were mostly low (Figure 2). Risk of bias assessment of individual studies is provided as Multimedia Appendix 3.

### Primary Outcomes: Clinical Effectiveness

**Figure 3:**
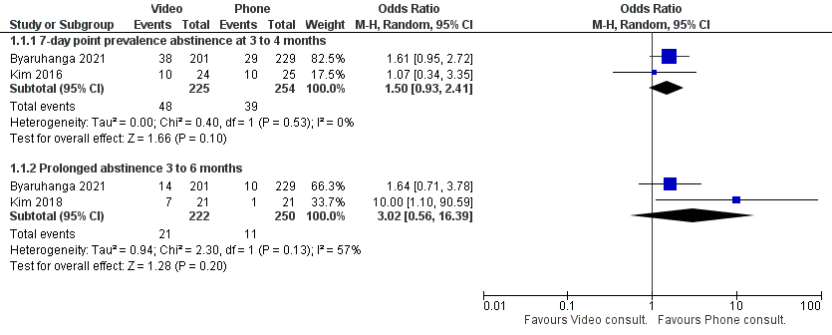
Difference in smoking abstinence rates between video and telephone consultations [19,31].

Three trials that were conducted in the community reported on smoking cessation outcomes [18,19,31]. Though the outcomes favored telephone consultations, overall, there was no significant difference between telephone and video interventions in smoking abstinence up to 6 months following interventions (Figure 3).

**Figure 4:**
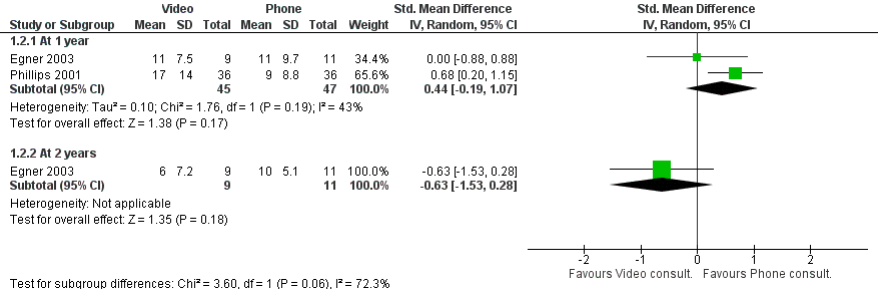
Difference in depression outcomes between video and telephone consultations [13,22].

For depression (measured by the Center for Epidemiological Studies Depression Scale score), 2 studies including patients with multiple sclerosis [13] and newly acquired spinal cord injury [22] found no significant difference in outcomes between telephone and video interventions up to 2 years (Figure 4).

**Figure 5:**
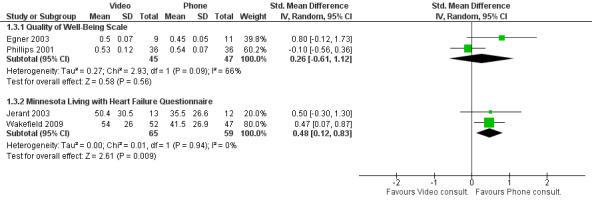
Difference in quality-of-life outcomes between video and telephone consultations [13,22].

Four studies reported quality-of-life outcomes [13,16,22,23]. There was no difference in quality of well-being scores between telephone and video interventions (Figure 5). However, patients in the telephone group scored a half a point more overall on the Minnesota Living with Heart Failure Questionnaire scores, which ranges between 0 to 105, higher scores indicating better quality of life. Although statistically significant, half a point is not likely to be clinically significant.

### Secondary Outcomes

#### Health Care Usage

Four studies in total reported on health care usage outcomes: 2 studies reported on the mean number of inpatient days [22,25], 1 study reported on total health care costs [17], and 1 study reported on both [26].

**Figure 6:**
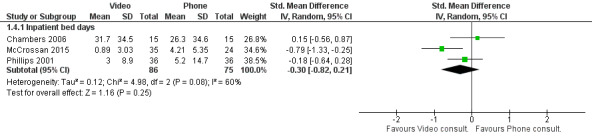
Difference in health care usage outcomes between video and telephone consultations [22,25,26].

Three studies reported outcomes associated with health care usage, specifically, inpatient days of the 2 intervention groups [22,25,26]. These study participants had either parenteral nutrition, congenital heart disease, or spinal cord injury and were monitored in the community. Overall, there was no significant difference between telephone and video intervention groups regarding the number of inpatient days (Figure 6). However, there was substantial heterogeneity, and notably, McCrossan et al’s [27] trial with children with congenital heart disease significantly favored video consultations.

Two other studies compared the total health care costs of the 2 intervention groups [17,26]. In a study with patients with chronic heart failure [17], the video care group’s total health care charges were higher than those of the telephone care group. Conversely, in a study with pediatric cardiology patients, the total health care costs were one-fourth those of the telephone care group [26]. However, in both studies, telephone and video interventions cost much less than usual care.

#### Satisfaction With Care

**Figure 7:**
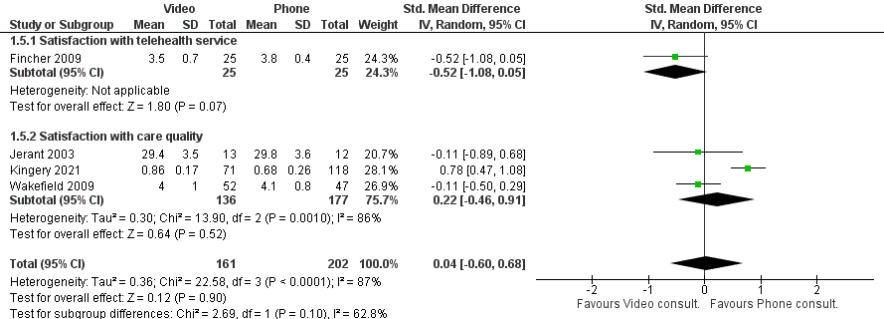
Patient satisfaction with telehealth [14].

Six studies reported on patient satisfaction with care, of which 3 are comparable and are shown in Figure 7 [14,16,23]. In the other 3 studies, the patients were equally satisfied with both telephone and video telehealth consultations in resolving their questions and concerns [20,27,28].

Seven studies addressed the acceptability and feasibility of the telehealth interventions [15,18,19,27-30]. Both telephone and video interventions were largely and equally acceptable; however, the main challenges for feasibility were access to video call equipment and individual patients’ condition severity and self-efficacy. Clinicians also found videoconferencing acceptable and were more confident in making clinical judgements via video calls rather than telephone calls [27,28].

## Discussion

### Principal Findings

This systematic review identified 16 RCTs, which provided a direct comparison of telephone and video telehealth consultations for ongoing care of patients with established diagnoses. There were no significant differences in clinical effectiveness, patient satisfaction, and health care use (cost-effectiveness) outcomes between the 2 modalities. Both telephone and video consultations were acceptable and feasible. Most of the studies had moderate to high risk of bias, thus reducing the quality of the evidence to low.

Many prior studies have demonstrated that telephone and video telehealth consultations by themselves can be equally safe and effective compared to face-to-face delivery with regard to acceptability, effectiveness, and safety outcomes for a wide variety of conditions such as diabetes [32,33] and mental health [5,34,35]. This review adds further evidence that telephone and video consultations are equally acceptable and effective when compared directly.

This review has several strengths. We developed and registered the protocol a priori, conducted rigorous search to find all available evidence, and followed PRISMA guidelines. Clear, strict inclusion and exclusion criteria allowed for studies in a variety of different health conditions to be synthesized and systematically reviewed. Furthermore, we only included RCTs and assessed the risk of bias of all included studies.

However, there are some limitations to our findings. All included studies were conducted in high- and middle-income countries and most included fewer than 50 participants, thus limiting the generalizability of our findings. Half of the studies were conducted prior to 2012 before smartphones were in widespread use, using special video calling devices installed in patients’ homes, which would limit the scalability of the intervention. However, with the increasing ownership of personal smartphones worldwide, video communications have become more accessible in recent years, and we believe that the findings from studies using older video calling devices would still be valid today in terms of clinical effectiveness. Lastly, as shown in the risk of bias assessment, some of the studies may have baseline inequalities due to unclear randomization processes; therefore, our results should be interpretated with caution.

The clinical relevance of our findings depends on the objectives and clinical contexts of the consultations. In most of our included studies, telehealth interventions were used to follow-up with patients with chronic conditions in the community. This demonstrates the value of telehealth to reduce unnecessary hospitalizations, ensure continuity of care, and improve accessibility of essential health care services for patients in rural and remote areas. Furthermore, different telehealth modalities may be better suited to different types of health care provisions. For example, clinicians were more confident in making clinical judgements and decisions via video rather than by telephone, but the patients were equally satisfied with both video and telephone consultations. However, there was a dearth of studies on telehealth consultations for diagnostic purposes. Future studies should address this gap and provide evidence for usability and effectiveness of telehealth interventions for diagnostic consultations.

Although the COVID-19 pandemic greatly accelerated the uptake of telehealth worldwide, we have not found any studies conducted in a primary care setting, which compared telephone to video consultations. Given the increase in convenience and accessibility and a decrease in health care costs, video or phone consultations could be highly beneficial in primary care delivery. Hence, there is an urgent need for studies in primary care settings comparing telephone to video delivery, to establish the most appropriate mode of health care delivery, particularly when access to face-to-face–delivered health care is limited by circumstances such as geography, disability, caretaking obligations, and limited health care resources.

### Conclusions

Based on 16 diverse trials, we found no notable differences between telephone and video consultations for the management of patients with an established diagnosis. However, many of the trials identified were small and old, and were conducted in high-income countries, thus limiting the generalizability of these conclusions. There is also a significant lack of telehealth research in primary care settings despite high uptake.

## Appendix

Multimedia Appendix 1 Full search strategies.

Multimedia Appendix 2 Excluded full text studies with reasons.

Multimedia Appendix 3 Risk of bias of individual studies.

Multimedia Appendix 4 PRISMA 2020 checklist.

## Abbreviations

PRISMA: Preferred Reporting Items for Systematic Reviews and Meta-Analyses
RCT: randomized controlled trial
