# Supplementary material for: Comparison of Telephone and Video Telehealth Consultations: Systematic Review
Source: J Med Internet Res. 2023 Nov 17;25:e49942. doi: 10.2196/49942 (PMC10692872; doi:10.2196/49942)
Supplement: Multimedia Appendix 1 [file jmir_v25i1e49942_app1.docx]

## Multimedia appendix of supplementary files

Appendix 1. Full search strategies

Appendix 2. Table of excluded studies with reasons

## Appendix 1. Search strategies

**RCT searches**

**PubMed**

("Telemedicine"[Mesh] OR Telehealth[tiab] OR Telemedicine[tiab] OR ((Telephone[tiab] OR Phone[tiab]) AND (Consultation[tiab] OR Consult[tiab])))

AND

("Primary Health Care"[Mesh] OR "General Practice"[Mesh] OR rehabilitation[sh] OR "Outpatients"[Mesh] OR "Speech Therapy"[Mesh] OR Outpatient[tiab] OR “Primary health”[tiab] OR “Primary care”[tiab] OR “General practice”[tiab] OR “General practices”[tiab] OR “General practitioners”[tiab] OR “General practitioner”[tiab] OR “Family practice”[tiab] OR Physician[tiab] OR Physicians[tiab] OR Clinician[tiab] OR Clinicians[tiab] OR Therapist[tiab] OR Nurse[tiab] OR Nurses[tiab] OR Physiotherapist[tiab] OR Rehabilitation[tiab] OR Diabetes[tiab] OR Diabetic[tiab] OR Asthma[tiab] OR Depression[tiab] OR “Ïrritable bowel”[tiab] OR IBS[tiab] OR PTSD[tiab] OR “Chronic fatigue”[tiab])

AND

("Videoconferencing"[Mesh] OR Videoconferencing[tiab] OR Videoconference[tiab] OR Videoconferences[tiab] OR Video[tiab] OR Teleconsultation[tiab] OR Skype[tiab] OR Zoom[tiab])

AND

("Delivery of Health Care"[Mesh] OR Delivery[tiab] OR Delivered[tiab] OR Via[tiab] OR Received[tiab])

AND

("Treatment Outcome"[Mesh] OR "Patient Satisfaction"[Mesh] OR Therapy[sh] OR Diagnosis[sh] OR “Clinical outcomes”[tiab] OR Treatment[tiab] OR Diagnostic[tiab] OR Efficacy[tiab])

AND

(Randomized controlled trial[pt] OR controlled clinical trial[pt] OR randomized[tiab] OR randomised[tiab] OR placebo[tiab] OR "drug therapy"[sh] OR randomly[tiab] OR trial[tiab] OR groups[tiab])

NOT

(Animals[Mesh] not (Animals[Mesh] and Humans[Mesh]))

NOT

(“Case Reports”[pt] OR Editorial[pt] OR Letter[pt] OR Meta-Analysis[pt] OR “Observational Study”[pt] OR “Systematic Review”[pt] OR “Case Report”[ti] OR “Case series”[ti] OR Meta-Analysis[ti] OR “Meta Analysis”[ti] OR “Systematic Review”[ti] OR “Systematic Literature Review”[ti] OR “Qualitative study”[ti] OR Protocol[ti])

**CENTRAL**

([mh Telemedicine] OR Telehealth:ti,ab OR Telemedicine:ti,ab OR ((Telephone:ti,ab OR Phone:ti,ab) AND (Consultation:ti,ab OR Consult:ti,ab)))

AND

([mh "Primary Health Care"] OR [mh "General Practice"] OR [mh Outpatients] OR [mh "Speech Therapy"] OR Outpatient:ti,ab OR "Primary health":ti,ab OR "Primary care":ti,ab OR "General practice":ti,ab OR "General practices":ti,ab OR "General practitioners":ti,ab OR "General practitioner":ti,ab OR "Family practice":ti,ab OR Physician:ti,ab OR Physicians:ti,ab OR Clinician:ti,ab OR Clinicians:ti,ab OR Therapist:ti,ab OR Nurse:ti,ab OR Nurses:ti,ab OR Physiotherapist:ti,ab OR Rehabilitation:ti,ab OR Diabetes:ti,ab OR Diabetic:ti,ab OR Asthma:ti,ab OR Depression:ti,ab OR "Ïrritable bowel":ti,ab OR IBS:ti,ab OR PTSD:ti,ab OR "Chronic fatigue":ti,ab)

AND

([mh Videoconferencing] OR Videoconferencing:ti,ab OR Videoconference:ti,ab OR Videoconferences:ti,ab OR Video:ti,ab OR Teleconsultation:ti,ab OR Skype:ti,ab OR Zoom:ti,ab)

AND

([mh "Delivery of Health Care"] OR Delivery:ti,ab OR Delivered:ti,ab OR Via:ti,ab OR Received:ti,ab)

AND

([mh "Treatment Outcome"] OR [mh "Patient Satisfaction"] OR "Clinical outcomes":ti,ab OR Treatment:ti,ab OR Diagnostic:ti,ab OR Efficacy:ti,ab)

**Embase**

('Telemedicine'/exp/mj OR Telehealth:ti,ab OR Telemedicine:ti,ab OR ((Telephone:ti,ab OR Phone:ti,ab) AND (Consultation:ti,ab OR Consult:ti,ab)))

AND

('Primary Health Care'/exp/mj OR 'General Practice'/exp/mj OR 'Outpatient'/exp/mj OR 'Speech Therapy'/exp/mj OR Outpatient:ti,ab OR "Primary health":ti,ab OR "Primary care":ti,ab OR "General practice":ti,ab OR "General practices":ti,ab OR "General practitioners":ti,ab OR "General practitioner":ti,ab OR "Family practice":ti,ab OR Physician:ti,ab OR Physicians:ti,ab OR Clinician:ti,ab OR Clinicians:ti,ab OR Therapist:ti,ab OR Nurse:ti,ab OR Nurses:ti,ab OR Physiotherapist:ti,ab OR Rehabilitation:ti,ab OR Diabetes:ti,ab OR Diabetic:ti,ab OR Asthma:ti,ab OR Depression:ti,ab OR "Ïrritable bowel":ti,ab OR IBS:ti,ab OR PTSD:ti,ab OR "Chronic fatigue":ti,ab)

AND

(Videoconferencing/exp/mj OR Videoconferencing:ti,ab OR Videoconference:ti,ab OR Videoconferences:ti,ab OR Video:ti,ab OR Teleconsultation:ti,ab OR Skype:ti,ab OR Zoom:ti,ab)

AND

('health care delivery'/exp/mj OR Delivery:ti,ab OR Delivered:ti,ab OR Via:ti,ab OR Received:ti,ab)

AND

('Treatment Outcome'/exp/mj OR 'Patient Satisfaction'/exp/mj OR "Clinical outcomes":ti,ab OR Treatment:ti,ab OR Diagnostic:ti,ab OR Efficacy:ti,ab)

AND

(random* OR factorial OR crossover OR placebo OR blind OR blinded OR assign OR assigned OR allocate OR allocated OR 'crossover procedure'/exp OR 'double-blind procedure'/exp OR 'randomized controlled trial'/exp OR 'single-blind procedure'/exp NOT ('animal'/exp NOT ('animal'/exp AND 'human'/exp)))

AND

[embase]/lim

**SR searches**

**PubMed**

("Telemedicine"[Mesh] OR Telehealth[tiab] OR Telemedicine[tiab] OR ((Telephone[tiab] OR Phone[tiab]) AND (Consultation[tiab] OR Consult[tiab])))

AND

("Primary Health Care"[Mesh] OR "General Practice"[Mesh] OR rehabilitation[sh] OR "Outpatients"[Mesh] OR "Speech Therapy"[Mesh] OR Outpatient[tiab] OR “Primary health”[tiab] OR “Primary care”[tiab] OR “General practice”[tiab] OR “General practices”[tiab] OR “General practitioners”[tiab] OR “General practitioner”[tiab] OR “Family practice”[tiab] OR Physician[tiab] OR Physicians[tiab] OR Clinician[tiab] OR Clinicians[tiab] OR Therapist[tiab] OR Nurse[tiab] OR Nurses[tiab] OR Physiotherapist[tiab] OR Rehabilitation[tiab] OR Diabetes[tiab] OR Diabetic[tiab] OR Asthma[tiab] OR Depression[tiab] OR “Ïrritable bowel”[tiab] OR IBS[tiab] OR PTSD[tiab] OR “Chronic fatigue”[tiab])

AND

("Videoconferencing"[Mesh] OR Videoconferencing[tiab] OR Videoconference[tiab] OR Videoconferences[tiab] OR Video[tiab] OR Teleconsultation[tiab] OR Skype[tiab] OR Zoom[tiab])

AND

("Delivery of Health Care"[Mesh] OR Delivery[tiab] OR Delivered[tiab] OR Via[tiab] OR Received[tiab])

AND

("Treatment Outcome"[Mesh] OR "Patient Satisfaction"[Mesh] OR Therapy[sh] OR Diagnosis[sh] OR “Clinical outcomes”[tiab] OR Treatment[tiab] OR Diagnostic[tiab] OR Efficacy[tiab])

AND

(Meta-Analysis[pt] OR “Systematic Review”[pt] OR Meta-Analysis[ti] OR “Meta Analysis”[ti] OR “Systematic Review”[ti] OR “Systematic Literature Review”[ti])

NOT

(“Case Reports”[pt] OR Editorial[pt] OR Letter[pt] OR “Observational Study”[pt] OR “Case Report”[ti] OR “Case series”[ti] OR “Qualitative study”[ti] OR Protocol[ti])

**CDSR via the Cochrane Library**

([mh Telemedicine] OR Telehealth:ti,ab OR Telemedicine:ti,ab OR ((Telephone:ti,ab OR Phone:ti,ab) AND (Consultation:ti,ab OR Consult:ti,ab)))

AND

([mh "Primary Health Care"] OR [mh "General Practice"] OR [mh Outpatients] OR [mh "Speech Therapy"] OR Outpatient:ti,ab OR "Primary health":ti,ab OR "Primary care":ti,ab OR "General practice":ti,ab OR "General practices":ti,ab OR "General practitioners":ti,ab OR "General practitioner":ti,ab OR "Family practice":ti,ab OR Physician:ti,ab OR Physicians:ti,ab OR Clinician:ti,ab OR Clinicians:ti,ab OR Therapist:ti,ab OR Nurse:ti,ab OR Nurses:ti,ab OR Physiotherapist:ti,ab OR Rehabilitation:ti,ab OR Diabetes:ti,ab OR Diabetic:ti,ab OR Asthma:ti,ab OR Depression:ti,ab OR "Ïrritable bowel":ti,ab OR IBS:ti,ab OR PTSD:ti,ab OR "Chronic fatigue":ti,ab)

AND

([mh Videoconferencing] OR Videoconferencing:ti,ab OR Videoconference:ti,ab OR Videoconferences:ti,ab OR Video:ti,ab OR Teleconsultation:ti,ab OR Skype:ti,ab OR Zoom:ti,ab)

AND

([mh "Delivery of Health Care"] OR Delivery:ti,ab OR Delivered:ti,ab OR Via:ti,ab OR Received:ti,ab)

AND

([mh "Treatment Outcome"] OR [mh "Patient Satisfaction"] OR "Clinical outcomes":ti,ab OR Treatment:ti,ab OR Diagnostic:ti,ab OR Efficacy:ti,ab)

**Embase**

('Telemedicine'/exp/mj OR Telehealth:ti,ab OR Telemedicine:ti,ab OR ((Telephone:ti,ab OR Phone:ti,ab) AND (Consultation:ti,ab OR Consult:ti,ab)))

AND

('Primary Health Care'/exp/mj OR 'General Practice'/exp/mj OR 'Outpatient'/exp/mj OR 'Speech Therapy'/exp/mj OR Outpatient:ti,ab OR "Primary health":ti,ab OR "Primary care":ti,ab OR "General practice":ti,ab OR "General practices":ti,ab OR "General practitioners":ti,ab OR "General practitioner":ti,ab OR "Family practice":ti,ab OR Physician:ti,ab OR Physicians:ti,ab OR Clinician:ti,ab OR Clinicians:ti,ab OR Therapist:ti,ab OR Nurse:ti,ab OR Nurses:ti,ab OR Physiotherapist:ti,ab OR Rehabilitation:ti,ab OR Diabetes:ti,ab OR Diabetic:ti,ab OR Asthma:ti,ab OR Depression:ti,ab OR "Ïrritable bowel":ti,ab OR IBS:ti,ab OR PTSD:ti,ab OR "Chronic fatigue":ti,ab)

AND

(Videoconferencing/exp/mj OR Videoconferencing:ti,ab OR Videoconference:ti,ab OR Videoconferences:ti,ab OR Video:ti,ab OR Teleconsultation:ti,ab OR Skype:ti,ab OR Zoom:ti,ab)

AND

('health care delivery'/exp/mj OR Delivery:ti,ab OR Delivered:ti,ab OR Via:ti,ab OR Received:ti,ab)

AND

('Treatment Outcome'/exp/mj OR 'Patient Satisfaction'/exp/mj OR "Clinical outcomes":ti,ab OR Treatment:ti,ab OR Diagnostic:ti,ab OR Efficacy:ti,ab)

AND

([cochrane review]/lim OR [systematic review]/lim OR [meta analysis]/lim OR ((Search:ti,ab OR Searched:ti,ab) AND (PubMed:ti,ab OR MEDLINE:ti,ab)) OR (Systematic:ti,ab AND Review:ti,ab) OR 'Meta analysis':ti,ab OR Meta-analysis:ti,ab OR Review:ti OR ((Systematically:ti,ab OR Reviewed:ti,ab) AND (literature:ti,ab)))

## Appendix 2. Excluded full text studies with reasons

| **No.** | **Excluded studies** | **Exclusion reason** |
| --- | --- | --- |
| 1 | Cady RG, Erickson M, Lunos S, et al. Meeting the needs of children with medical complexity using a telehealth advanced practice registered nurse care coordination model. Matern Child Health J 2015; 19(7): 1497-506. | Not telephone vs video comparison |
| 2 | Choi NG, Marti CN, Bruce ML, Hegel MT, Wilson NL, Kunik ME. Six-month postintervention depression and disability outcomes of in-home telehealth problem-solving therapy for depressed, low-income homebound older adults. Depress Anxiety 2014; 31(8): 653-61. | Interventions not comparable (not similar enough conditions) |
| 3 | Franek J. Home telehealth for patients with chronic obstructive pulmonary disease (COPD): an evidence-based analysis. Ont Health Technol Assess Ser 2012; 12(11): 1-58. | Not a RCT |
| 4 | Franklin CL, Cuccurullo LA, Walton JL, Arseneau JR, Petersen NJ. Face to face but not in the same place: A pilot study of prolonged exposure therapy. J Trauma Dissociation 2017; 18(1): 116-30. | Not telephone vs video comparison |
| 5 | Gallagher-Thompson D, Wang PC, Liu W, et al. Effectiveness of a psychoeducational skill training DVD program to reduce stress in Chinese American dementia caregivers: results of a preliminary study. Aging Ment Health 2010; 14(3): 263-73. | Not telephone vs video comparison |
| 6 | Gunasekeran DV, Liu Z, Tan WJ, et al. Evaluating Safety and Efficacy of Follow-up for Patients With Abdominal Pain Using Video Consultation (SAVED Study): Randomized Controlled Trial. J Med Internet Res 2020; 22(6): e17417. | Interventions not comparable (not similar enough conditions) |
| 7 | Meyer BC, Raman R, Ernstrom K, et al. Assessment of long-term outcomes for the STRokE DOC telemedicine trial. J Stroke Cerebrovasc Dis 2012; 21(4): 259-64. | Clinician-to-clinician telehealth |
| 8 | Meyer BC, Raman R, Hemmen T, et al. Efficacy of site-independent telemedicine in the STRokE DOC trial: a randomised, blinded, prospective study. Lancet Neurol 2008; 7(9): 787-95. | Clinician-to-clinician telehealth |
| 9 | Richter KP, Shireman TI, Ellerbeck EF, et al. Comparative and cost effectiveness of telemedicine versus telephone counseling for smoking cessation. Journal of medical Internet research 2015; 17(5): e113. | Interventions not comparable (not similar enough conditions) |
| 10 | Richter KP, Shireman TI, Ellerbeck EF, et al. Comparative and cost effectiveness of telemedicine versus telephone counseling for smoking cessation. Journal of medical Internet research 2015; 17(5): e113. | Duplicate |
| 11 | Voils CI, Venne VL, Weidenbacher H, Sperber N, Datta S. Comparison of Telephone and Televideo Modes for Delivery of Genetic Counseling: a Randomized Trial. J Genet Couns 2018; 27(2): 339-48. | Interventions not comparable (not similar enough conditions) |
| 12 | Wong HT, Poon WS, Jacobs P, et al. The comparative impact of video consultation on emergency neurosurgical referrals. Neurosurgery 2006; 59(3): 607-13; discussion -13. | Clinician-to-clinician telehealth |
