# Supplementary material for: Comparison of Telephone and Video Telehealth Consultations: Systematic Review
Source: J Med Internet Res. 2023 Nov 17;25:e49942. doi: 10.2196/49942 (PMC10692872; doi:10.2196/49942)
Supplement: Multimedia Appendix 2 [file jmir_v25i1e49942_app2.docx]

Multimedia appendix 2. Excluded full text studies with reasons

| **No.** | **Excluded studies** | **Exclusion reason** |
| --- | --- | --- |
| 1 | Cady RG, Erickson M, Lunos S, et al. Meeting the needs of children with medical complexity using a telehealth advanced practice registered nurse care coordination model. Matern Child Health J 2015; 19(7): 1497-506. | Not telephone vs video comparison |
| 2 | Choi NG, Marti CN, Bruce ML, Hegel MT, Wilson NL, Kunik ME. Six-month postintervention depression and disability outcomes of in-home telehealth problem-solving therapy for depressed, low-income homebound older adults. Depress Anxiety 2014; 31(8): 653-61. | Interventions not comparable (not similar enough conditions) |
| 3 | Franek J. Home telehealth for patients with chronic obstructive pulmonary disease (COPD): an evidence-based analysis. Ont Health Technol Assess Ser 2012; 12(11): 1-58. | Not a RCT |
| 4 | Franklin CL, Cuccurullo LA, Walton JL, Arseneau JR, Petersen NJ. Face to face but not in the same place: A pilot study of prolonged exposure therapy. J Trauma Dissociation 2017; 18(1): 116-30. | Not telephone vs video comparison |
| 5 | Gallagher-Thompson D, Wang PC, Liu W, et al. Effectiveness of a psychoeducational skill training DVD program to reduce stress in Chinese American dementia caregivers: results of a preliminary study. Aging Ment Health 2010; 14(3): 263-73. | Not telephone vs video comparison |
| 6 | Gunasekeran DV, Liu Z, Tan WJ, et al. Evaluating Safety and Efficacy of Follow-up for Patients With Abdominal Pain Using Video Consultation (SAVED Study): Randomized Controlled Trial. J Med Internet Res 2020; 22(6): e17417. | Interventions not comparable (not similar enough conditions) |
| 7 | Meyer BC, Raman R, Ernstrom K, et al. Assessment of long-term outcomes for the STRokE DOC telemedicine trial. J Stroke Cerebrovasc Dis 2012; 21(4): 259-64. | Clinician-to-clinician telehealth |
| 8 | Meyer BC, Raman R, Hemmen T, et al. Efficacy of site-independent telemedicine in the STRokE DOC trial: a randomised, blinded, prospective study. Lancet Neurol 2008; 7(9): 787-95. | Clinician-to-clinician telehealth |
| 9 | Richter KP, Shireman TI, Ellerbeck EF, et al. Comparative and cost effectiveness of telemedicine versus telephone counseling for smoking cessation. Journal of medical Internet research 2015; 17(5): e113. | Interventions not comparable (not similar enough conditions) |
| 10 | Richter KP, Shireman TI, Ellerbeck EF, et al. Comparative and cost effectiveness of telemedicine versus telephone counseling for smoking cessation. Journal of medical Internet research 2015; 17(5): e113. | Duplicate |
| 11 | Voils CI, Venne VL, Weidenbacher H, Sperber N, Datta S. Comparison of Telephone and Televideo Modes for Delivery of Genetic Counseling: a Randomized Trial. J Genet Couns 2018; 27(2): 339-48. | Interventions not comparable (not similar enough conditions) |
| 12 | Wong HT, Poon WS, Jacobs P, et al. The comparative impact of video consultation on emergency neurosurgical referrals. Neurosurgery 2006; 59(3): 607-13; discussion -13. | Clinician-to-clinician telehealth |
