# Supplementary figures and images for: Comparison of Telephone and Video Telehealth Consultations: Systematic Review
Source: J Med Internet Res. 2023 Nov 17;25:e49942. doi: 10.2196/49942 (PMC10692872; doi:10.2196/49942)

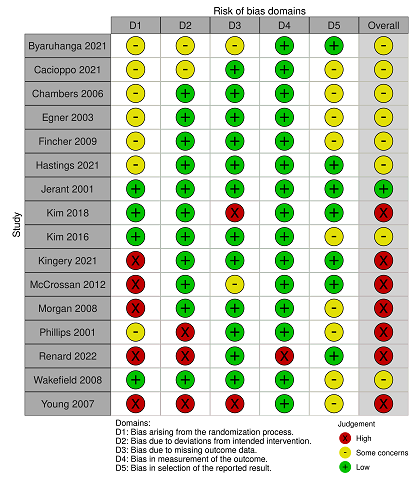

Supplement: Multimedia Appendix 3 [file jmir_v25i1e49942_app3.png]
